# Concomitant Infection of *Helicobacter pylori* and Intestinal Parasites: Burden, Sociodemographic and Clinical Characteristics in Hospitalized Children and Adolescents in Northern Lebanon

**DOI:** 10.12688/f1000research.148550.1

**Published:** 2024-05-17

**Authors:** Sara MINA, Sara Daher, Nour Mina, Ghalia Khoder

**Affiliations:** 1 Department of Medical Laboratory Sciences, Faculty of Health Sciences, Beirut Arab University, Beirut, 11-5020, Lebanon; 2 Faculty of Public Health 3, L.S.E.E, Lebanese University, Tripoli, Lebanon; 3 Faculty of Medicine, Beirut Arab University, Beirut, 11-5020, Lebanon; 4 Department of Pharmaceutics and Pharmaceutical Technology, College of Pharmacy, University of Sharjah, Sharjah, 27272, United Arab Emirates; 5 Sharjah Institute for Medical Research, University of Sharjah, Sharjah, Sharjah, 27272, United Arab Emirates

**Keywords:** Co-infection, Lebanon, Intestinal parasites, Helicobacter pylori, Stool.

## Abstract

**Background:**

*Helicobacter pylori* and intestinal parasites are well-known for their high prevalence in children, especially in developing countries. However, their concomitant infections are poorly documented. In this study, we aimed to evaluate the association between intestinal parasites and *H. pylori* among hospitalized children and adolescents with upper gastrointestinal complaints in Northern Lebanon.

**Methods:**

A cross-sectional study was conducted involving 297 hospitalized pediatric patients, aged between 1 and 15 years, who presented with gastrointestinal symptoms. The socio-demographic, lifestyle, and gastrointestinal characteristics of all participants were analyzed. Fresh stool samples were collected and screened for the presence of intestinal parasites and *H. pylori* infections.

**Results:**

6.4% of the patients were positive for intestinal parasitic infections, 5.4% were positive for *H. pylori* infection, and 11.8% were co-infected. The results of the Chi-square test showed that *H. pylori* infection is significantly associated with parasitic infection but not with a particular species. The most frequent coinfection was *H. pylori-Entamoeba histolytica* (77.1%). Moreover, *H. pylori* infection was associated with overcrowding and infrequent washing of vegetables before eating. The prevalence of co-infections increased in patients of mothers with a primary educational level or less. In regards to clinical characteristics, our findings showed a statistically significant relationship between i) gastric reflux and *H. pylori*, and ii) severe diarrhea and parasitic infection.

**Conclusion:**

Our data highlighted the association between *H. pylori* and intestinal parasitic infections. Thus, *H. pylori* detection could be taken into consideration while screening for parasitic infections in children and adolescents.

## Introduction

Gastrointestinal symptoms are common in the pediatric population, especially in developing countries. Co-infections involving bacteria and protozoa are an emerging phenomenon featuring concurrent ecological niches or shared transmission routes and risk factors ( Akoolo et al., 2022). Consequently, co-infecting pathogens may interplay synergistically or antagonistically and modulate disease severity and host health outcomes ( Hoarau et al., 2020; Devi et al., 2021). Intestinal parasites and the pathobiont bacterium *Helicobacter pylori* ( *H. pylori*) are well known for their high prevalence in children, especially in low-resource settings.

*H. pylori* infection has been extensively investigated over the last 30 years. It may currently be the most well-studied chronic bacterial infection in humans in a specific biological niche, specifically in the gut territory, and one of the major public health problems ( Hooi et al., 2017). *H. pylori* colonization is one of the most prevalent infectious diseases and is predominantly acquired during childhood. A higher burden was reported in developing countries at a younger age than in developed countries ( Borka Balas et al., 2022). Despite the vast number of studies that have been conducted to extensively investigate the spread of *H. pylori*, its main route of transmission is still elusive and no predominant mode has been yet identified. It is widely believed that person-to-person transmission is the most common mode of transmission. Moreover, it has been proven that *H. pylori* can be transmitted vertically or horizontally by three possible pathways: the fecal-oral, the oral-oral, and the gastro-oral routes ( Kayali et al., 2018). Of note, recently published data pointed out the probable role of untreated water and food products in the environmental transmission of *H. pylori* ( Vesga et al., 2023). However, the scarcity of data failed to ascribe a definite foodborne transmission, thus extensive epidemiological studies should be performed to corroborate this hypothesis. Clinical manifestations are non-specific but some may be alarming in children such as persistent abdominal pain, vomiting, and gastrointestinal bleeding that require histopathologic examination of *H. pylori* ( Aguilera Matos et al., 2020). Gastric reflux is often associated with *H. pylori* infection even without the presence of macroscopic lesions on gastroduodenal mucosa ( Burkitt et al., 2017).

Intestinal parasites are significant contributors to morbidity and mortality in the pediatric population. Their prevalence is significantly associated with poor sanitation and fecal contamination of water supplies ( Hernández-Castro et al., 2024). Major burdens of intestinal parasitic infections in children are attributed to members of the *Entamoeba* complex ( *Entamoeba histolytica (E. histolytica)*, *Entamoeba dispar* and *Entamoeba moshkovskii*), *Blastocystis hominis (B. hominis)*, *Giardia lamblia (G. lamblia)* (syn. *Giardia intestinalis* and *Giardia duodenalis*), and *Cryptosporidium.* Their transmission typically occurs through the fecal-oral route after direct or indirect contact (by contaminated food or water) with the infective forms (cysts/oocysts) ( Ahmed, 2023). In Lebanon, recent studies reported cases of *E. histolytica* infection in South Lebanon and Beirut ( Salami et al., 2019; El Achkar et al., 2023). Other protozoa including *B. hominis*, *Entamoeba coli (E. coli)*, and *G. lamblia* were also found but at lower prevalence ( El Achkar et al., 2023).

Several previous studies from different locations have highlighted a potential association between intestinal parasites and *H. pylori* ( Ankarklev et al., 2012; Seid et al., 2018; Ibrahim et al., 2019). Since these pathogens are likely to share similar routes of transmission and risk factors, it is crucial to investigate this type of co-infection in the pediatric population. Not much data is available in Lebanon about the prevalence of *H. pylori* in this population and its potential association with intestinal parasitic infections. Understanding the prevalence pattern of *H. pylori* and intestinal parasite co-infections in the Lebanese pediatric population and their risk factors will aid in prioritizing public health efforts to better manage the burden of these infections and long-term complications.

## Methods

### Enrollment procedure and data collection

The sample size was calculated using the sample size calculator ( https://select-statistics.co.uk/calculators/sample-size-calculator-population-proportion/) with a confidence level of 95%, a margin of error of 5%, and an estimated prevalence of 25% based on a previous local study ( Khoder et al., 2021). The suggested sample size was initially 289 participants; however, 297 participants were included to account for any equivocal data that may be removed during the study. Patients eligible for the study consisted of any hospitalized child or adolescent presenting with complaints of one or more upper gastrointestinal symptoms at one of the two healthcare facilities during the study period. Pediatric patients met the following inclusion criteria: (i) aged less than 15 years old, (ii) onset of symptoms within the last week. An informed consent statement was signed by the patient or his parents. Participants were excluded if they i) had chronic diarrhea, ii) had a documented history of non-infectious etiology (symptoms persisting for longer than 4 weeks), iii) had recent antiparasitic therapy or proton pump inhibitors and bismuth preparations within the last two weeks before study enrollment, and iv) had viral diarrhea. Each patient or legal representative completed a face-to-face questionnaire addressing sociodemographic and socioeconomic characteristics, lifestyle, and clinical characteristics (presence of gastrointestinal symptoms).

### Sample collection and microbiological analysis

A stool specimen was collected from each patient before any prescribed antimicrobial therapy. Fresh stool samples were labeled and received in an airtight transport container and divided into two parts. One to two milligrams were immediately examined macroscopically for color and consistency. In addition, the presence of whole or partial parts of parasites was investigated using wet mount preparation in normal saline by direct-light microscopy (DLM). All examinations were repeated twice by two experienced microbiologists. The remaining stool samples were stored either at 4°C for up to three days or immediately frozen at −20°C until tested for the presence of *H. pylori* antigen. *H. pylori* infection was detected from patients’ fecal specimens by the *H. PYLORI QUICK* CHEK ^TM^ kit (TECHLAB, USA), a rapid qualitative sandwich enzyme immunoassay that uses immobilized capture monoclonal anti- *H. pylori* antibodies. Diluted fecal samples and controls were added to each antibody-coated microtiter well. Tests were performed in duplicate according to the manufacturer’s instructions. Absorbance equal to or above 0.12 at 450 nm or 0.08 at 450/620 nm or 450/630 nm was considered a positive result.

### Statistical analysis

Data were entered and statistical analyses were performed using the Statistical Package for the Social Sciences (SPSS) program (version 21.0) ( https://www.ibm.com/products/spss-statistics). Descriptive statistics were reported as frequencies and percentages for the categorical variables and as means and standard deviations (SD) for the continuous variables. The differences in gastrointestinal symptoms, sociodemographic characteristics, and hygiene-related practices between study groups were examined using the Chi-square test for categorical variables and independent t-tests for continuous variables. A *p* ≤ 0.05 was considered statistically significant.

## Results

### Sociodemographic and lifestyle characteristics

The sociodemographic characteristics of 297 children and adolescents included in this study are presented in Table 1. Patients aged less than 5 years constituted 68.0% of the study sample, followed by 23.9% aged 5-10 years, and 8.1% aged 10-15 years. Most of the patients were residing in urban areas (79.8%). In addition, 61.5% and 80.5% of mothers and fathers respectively, had an educational level of primary school or less. Furthermore, 83.5% of the study sample households reported having an insufficient monthly income.

**Table 1.** Sociodemographic characteristics of study participants (N = 297).

Variable	n (%)
**Gender**	
Male	155 (52.2)
Female	142 (47.8)
**Age in years**	
Less than 5	202 (68.0)
5-10	71 (23.9)
10-15	24 (8.1)
**Residence**	
Rural	60 (20.2)
Urban	237 (79.8)
**Mother’s education**	
Primary or less	183 (61.6)
Secondary or higher	114 (38.4)
**Father’s education**	
Primary or less	239 (80.5)
Secondary or higher	58 (19.5)
**Monthly income sufficiency**	
Sufficient	49 (16.5)
Not sufficient	248 (83.5)

### Assessment of *H. pylori* infection, intestinal parasitic infections, and *H. pylori* and parasitic co-infection

Patients were divided into four groups according to their infection status: i) intestinal parasitic infection only (IP infection), ii) *H. pylori* infection only (HP infection), iii) *H. pylori* and intestinal parasitic co-infection (HP-IP co-infection), and iv) *H. pylori* and intestinal parasites free infections (Free Infection).

Among the 297 tested patients’ stools, 6.4% were positive for IP infection, 5.4% were positive for HP infection, 11.8% were positive for HP-IP co-infection, and 76.4% were negative (free infection). Furthermore, *E. histolytica* accounted for the highest proportion of parasitic infection (77.8%), followed by *G. lambliae* (20.4%), and *E. coli* (1.9%). However, no statistical significance between the type of parasite and HP-IP co-infection was observed ( Table 2). Furthermore, when diagnosed with HP infection, a higher percentage of patients exhibited a positive mixed parasitic infection compared to cases where *H. pylori* infection was absent (66.0% vs. 35.2%, *p* < 0.001) ( Table 2).

**Table 2.** Prevalence of *H. pylori* and intestinal parasites among study participants.

	*H. pylori* infection n(%)		Total n(%)	p-value
**Parasitic infection**	Negative	Positive		
Parasitic infection (-)	227 (93.4)	16 (6.6)	243 (100)	**<0.001**
Parasitic infection (+)	19 (35.2)	35 (66.0)	54 (100)	
Total			297 (100)	
**Parasitic species**				
*Giardia lamblia*	4 (21.1)	7 (20.0)	11 (20.4)	0.267 *
*Entamoeba histolytica*	15 (78.9)	27 (77.1)	42 (77.8)	
*Entamoeba coli*	0 (0.0)	1 (2.9)	1 (1.9)	
Total	19 (100)	35 (100)	54 (100)	

* Cells have expected count less than 5 – used Fisher’s test instead of Pearson Chi-Square.

### Assessment of the clinical manifestations among the different groups

**Figure 1.**
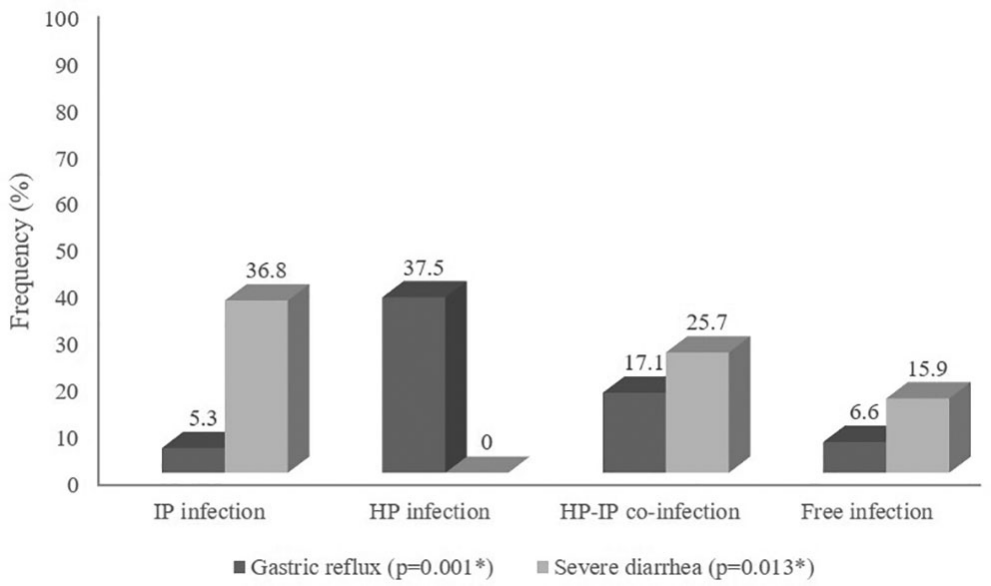
Comparative assessment of gastric reflux and severe diarrhea percentages among the different groups (%), (N = 297).

The most frequently reported symptoms among children and adolescents were vomiting (56.6%), abdominal pain (39.4%), mild diarrhea (33.3%), and fever (28.3%) with no statistically significant differences between study groups ( *p* > 0.005) as observed in Table 3 and Figure 1. On the other hand, children infected with *H. pylori* only or co-infected reported higher frequencies of reflux symptom (37.5% and 17.1% respectively) than those with intestinal parasitic infection only (5.3%) or without infection (6.6%) ( *p* = 0.001). However, patients infected with *H. pylori* alone or free of both infections had zero or lower percentages of severe diarrhea (0.0% and 15.9% respectively) compared to those infected with intestinal parasitic infections (36.8%) or both *H. pylori* and intestinal parasitic infections (25.7%) with a statistically significant difference between the study infectious groups ( *p *= 0.013).

**Table 3.** Comparative assessment of the clinical manifestations among the different groups (n, %), (N = 297).

Clinical manifestation	IP infection (n = 19)	HP infection (n = 16)	HP-IP co-infection (n = 35)	Free infection (n = 227)	Total (n = 297)	*p*-value
**Bloating**	0 (0.0)	0 (0.0)	0 (0.0)	21 (9.3)	21 (7.1)	0.100 *
**Nausea**	2 (10.5)	1 (6.3)	3 (8.6)	33 (14.5)	39 (13.1)	0.769 *
**Vomiting**	14 (73.7)	7 (43.8)	21 (60.0)	126 (55.5)	168 (56.6)	0.307
**Abdominal pain**	9 (47.4)	5 (31.3)	11 (31.4)	92 (40.5)	117 (39.4)	0.572
**Gastric reflux**	1 (5.3)	6 (37.5)	6 (17.1)	15 (6.6)	28 (9.4)	**0.001** *
**Loss of appetite**	0 (0.0)	0 (0.0)	0 (0.0)	2 (0.9)	2 (0.7)	1.000 *
**Fever**	9 (47.4)	4 (25.0)	7 (20.0)	64 (28.2)	84 (28.3)	0.210 *
**Mild diarrhea**	11 (57.9)	7 (43.8)	16 (45.7)	86 (37.9)	99 (33.3)	0.321
**Severe diarrhea**	7 (36.8)	0 (0.0)	9 (25.7)	36 (15.9)	54 (18.2)	**0.013** *

* Cells have expected count less than 5 – used Fisher’s test instead of Pearson Chi-Square.

### Association of risk factors with intestinal parasitic infection, *H. pylori* infection, and *H. pylori*-parasitic co-infections

Table 4 summarizes the associations between the four study groups and the sociodemographic and hygiene-related practices. Children and adolescents residing in urban areas exhibited a higher susceptibility to both mono-infections and mixed infections by intestinal parasites and *H. pylori* in comparison to their rural counterparts ( *p* = 0.453). Additionally, a significantly elevated crowding index was observed among patients with HP infection when compared to other groups ( *p* = 0.025).

**Table 4.** Sociodemographic characteristics and hygiene-related practices association with the different study groups (n, %) (N = 297).

	IP infection n (%)	HP infection n (%)	HP-IP Co-infection n (%)	Free infection n (%)	*p*-value
**Sociodemographic characteristics**					
**Age group**					
<5 years	12 (63.2%)	12 (75.0%)	17 (48.6%)	161 (70.9%)	0.119 *
5-10 years	6 (31.6%)	2 (12.5%)	13 (37.1%)	50 (22.0%)	
10-15 years	1 (5.3%)	2 (12.5%)	5 (14.3%)	16 (7.0%)	
**Gender**					
Male	9 (47.4%)	6 (37.5%)	20 (57.1%)	120 (52.9%)	0.584 *
Female	10 (52.6%)	10 (62.5%)	15 (42.9%)	107 (47.1%)	
**Residency**					
Rural	2 (10.5%)	2 (11.1%)	5 (12.5%)	51 (18.3%)	0.453 *
Urban	17 (89.5%)	16 (88.9%)	35 (87.5%)	227 (81.7%)	
**Mother’s education**					
Primary or less	13 (68.4)	11 (68.8)	29 (82.9)	129 (57.1)	**0.025** *
Secondary or higher	6 (31.6)	5 (31.3)	6 (17.1)	97 (42.9)	
**Father’s education**					
Primary or less	15 (78.9)	16 (100.0)	31 (88.6)	177 (78.0)	0.078
Secondary or higher	4 (21.1)	0 (0.0)	4 (11.4)	50 (22.0)	
**Monthly income**					
Sufficient	4 (21.1)	1 (6.3)	5 (14.3)	39 (17.2)	0.697
Not sufficient	15 (78.9)	15 (93.8)	30 (85.7)	188 (82.8)	
**Crowding index** (Mean ± SD)	2.5±1.2	3.6±1.9	2.9±1.3	2.5±1.5	**0.025**
**Hygiene-related practices**					
**Water source**					
Treated	9 (47.4)	3 (18.8)	11 (31.4)	96 (42.5)	0.166
Untreated	10 (52.6)	13 (81.3)	24 (68.6)	130 (57.5)	
**Wash vegetables before eating**					
Always	16 (84.2)	11 (68.8)	28 (82.4)	208 (92.0)	**0.010** *
Sometimes	3 (15.8)	3 (18.8)	6 (17.6)	14 (6.2)	
Never	0 (0.0)	2 (12.5)	0 (0.0)	4 (1.8)	
**Wash hands before meals**					
Always	13 (68.4)	10 (66.7)	25 (73.5)	157 (72.4)	0.690 *
Sometimes	6 (31.6)	3 (20.0)	8 (23.5)	46 (21.2)	
Never	0 (0.0)	2 (13.3)	1 (2.9)	14 (6.5)	
**Wash hands after defecating**					
Correct way	12 (92.3)	8 (72.7)	20 (80.0)	114 (76.0)	**0.046** *
Incorrect way	1 (7.7)	3 (27.3)	2 (8.0)	7 (4.7)	
Do not wash hands	0 (0.0)	0 (0.0)	3 (12.0)	29 (19.3)	

* Cells have expected count less than 5 – used Fisher’s test instead of Pearson Chi-Square.

Children and adolescents of mothers with primary education or lower exhibited a significantly elevated likelihood of HP-IP compared to those born to mothers with secondary education or higher ( *p* = 0.025). Furthermore, patients from households that reported never washing vegetables before eating were significantly more likely to be infected with *H. pylori* only ( *p* = 0.010). Surprisingly, children and adolescents from households that reported washing hands correctly after defecating were significantly more likely infected with intestinal parasites only ( *p* = 0.046).

## Discussion

Gastrointestinal complaints including gastric reflux, diarrhea, and vomiting are one of the major indications for pediatric hospital admissions ( Dipasquale et al. 2018). *H. pylori*, *E. histolytica*, and *G. lamblia* are the most common intestinal pathogens in humans residing in low- and middle-income countries. In the present study, the overall prevalence of intestinal parasitic infection was found to be 54%, among them *E. histolytica* was the predominant protozoan parasite (77.8%). It is worth noting that our findings indicate that the North Lebanese pediatric population were at higher risk of intestinal parasitic infections than their counterparts admitted to a private tertiary care hospital located in South Lebanon (26.3%) ( Ghssein et al., 2018). In addition, a recent retrospective study addressed the prevalence of intestinal parasites in all age groups of patients at a tertiary care center in Beirut where *E. histolytica* accounted for 7% and 10% of the cases during the pre-COVID and post-COVID periods, respectively ( El Achkar et al., 2023). Although these three governorates belong to the same biogeographic region and share similar geographical and climatic conditions, the observed discrepancies could be explained by intrinsic differences in sanitation infrastructures and facilities. Moreover, this difference can be also associated with compounded crises following the deteriorated economic condition, and most importantly the Syrian influx that is greatly pronounced in the Northern region of Lebanon.

Our findings showed an increase in the prevalence of *H. pylori* infection with overcrowding and infrequent washing of vegetables before eating. These factors may facilitate the transmission of *H. pylori* within households and support the intra-familial clustering of this pathogen ( Palanduz et al., 2018). These findings were consistent with recent reports from different countries where living in crowded households was found to be a significant risk factor for *H. pylori* infection ( Aitila et al., 2019; Che et al., 2022). Moreover, our findings support and extend evidence reported in several studies worldwide regarding the transmission of *H. pylori* through unwashed vegetables ( Szaflarska-Popławska and Soroczyńska-Wrzyszcz, 2019; Yisak et al., 2022). This can be explained by the direct contamination of vegetables by the bacterium or by irrigation with untreated wastewater ( Kayali et al., 2018). In addition, vegetables may provide a suitable niche for *H. pylori* survival thanks to the ability of this bacteria to form biofilm and microcolonies on vegetable surfaces for an extended period ( Ng et al., 2017). Thus, vegetables may potentially serve as a transmission vehicle for *H. pylori,* however, this hypothesis is still poorly documented.

Intestinal parasites and *H. pylori* share similar predisposing factors, thus a better understanding of the epidemiology of *H. pylori* infection in children infected with intestinal parasites is crucial to investigate whether *H. pylori* provides a favorable niche for intestinal parasitic infections or vice versa. We did not find any statistical significance between HP and a specific IP infection, so our analysis was based on HP-IP co-infections. Our findings showed that higher *H. pylori* colonization was found among individuals infected with intestinal parasites than non-infected subjects. The most frequent intestinal parasites were protozoa, and specifically, the association of *H. pylori* with *E. histolytica* was the most prevalent. Comparative data highlighting these co-infections were reported in African countries. A similar *E. histolytica* and *H. pylori* co-infection (12%) was reported in a Sudanese study that included individuals from all age groups ( Yousif Abd Elbagi et al., 2019). Another cross-sectional study enrolled children aged 14 years or younger living in Ethiopia and showed a 23% prevalence of coinfection with any intestinal parasite and *H. pylori.* Maternal education was described as a significant risk factor for co-infection ( Spotts et al., 2020). Abd El Hameed et al. (2021) showed an association between *H. pylori* and parasitic infections, especially *G. lamblia* (35.9%), in Egyptian children aged between 1 and 15 years. In Europe, a single study conducted in Northeastern Italy found a significant association between *H. pylor*i and concomitant parasitic infection in different ethnicities, with *Blastocystis* being the most frequent co-infecting parasite ( Pomari et al., 2020). Fuenmayor-Boscán et al. (2016) reported a significant association with helminths in South American children aged less than 5 years old. Nonetheless, their finding disagreed with ours that showed an inverse association between *H. pylori* and *G. lamblia*, indicating an antagonistic interaction that may hinder the colonization of the gut by exogenous intestinal pathogens. These associations detected in different countries support the similar fecal-oral route in the transmission of the etiologic agents. However, the environmental exposure factors to the fecal-oral transmission route investigated herein were associated with *H. pylori* only regarding the infrequency of washing vegetables before eating ( *p* = 0.01) and handwashing with soap after defecating ( *p* = 0.046). Regarding the epidemiological factors associated with co-infections, children and adolescents of mothers with primary education or less were significantly more likely to be co-infected compared with children from mothers with higher educational levels ( *p* = 0.025). It is noteworthy that the homogenous lifestyle of this study population may have masked any possible association between this environmental factor and intestinal infections or co-infections. Moreover, urease production by *H. pylori* converts urea into carbon dioxide and ammonia resulting in buffering the environment and thus enhancing bacterial colonization. Changes in the local environment could result in a higher incidence of intestinal parasitic infections by modulating the host’s immune responses. Furthermore, *H. pylori* was described as a candidate training of immune cells, especially primary human monocytes, towards subsequent microbial pathogens ( Frauenlob et al., 2023).

Vomiting was the most frequent complaint (60%) in co-infected children, and in patients infected with intestinal parasites (73.7%), while it was less frequently recorded in *H. pylori-*infected and in co-infected individuals (43.8% and 60%, respectively). In addition, gastric reflux was significantly associated (37.5%; *p* = 0.001) with *H. pylor*i infection. On the other hand, severe diarrhea was less recorded in co-infected children (24.7%) than in those with intestinal parasitic infection (36.8%; *p* = 0.013). Consequently, co-infection could modulate the host immune response and, thus, the clinical manifestation of each pathogen regarding severe diarrhea. Indeed, it has been described that children who tested positive for *H. pylori* are at lower risk of diarrheal diseases, especially during giardiasis, than children who are negative for this infection ( Abd El Hameed et al., 2021). These results suggest that pediatric infections with *H. pylori* may condition a balanced host immune response to subsequent infections by playing a potential protective role during the diarrheal episode in co-infected patients and suppressing hyperimmune responses in the gut. Previous investigations seem to support this argument. Interferon-gamma (IFN-γ) is secreted along with Th1 responses toward intestinal amebiasis and was shown to play a protective role by clearing the amebae ( Deloer et al., 2017). It can be also expressed at a higher level in *H. pylori*-positive children than in healthy children ( Yu et al., 2020). Thus, patients enrolled in our study can be at the early stage of amebiasis and IFN-γ may reduce the risk of severe outcomes in *H. pylori*-positive individuals. However, our findings are not consistent with previous reports that demonstrated that the presence of intestinal protozoa in the gut together with *H. pylori* amplifies the recruitment of Th1 cells. This leads to exaggerated inflammation and tissue damage and thus it may worsen the disease ( Krzyżek and Gościniak, 2017).

The present study is the first to report novel assessment and risk data on the association between *H. pylori* and intestinal parasites in the Lebanese pediatric population attending tertiary care hospitals. Future large-scale and multicenter epidemiological studies should be performed using different diagnostic methods to support these findings and to explore the mechanistic interaction between *H. pylori* and particular types of parasitic infection. Understanding these interactions highlights the importance of considering and mitigating these co-infections by health authorities and decision-makers to improve the methods of detection and implement effective intervention measures and public health strategies.

## Conclusion

The association between *H. pylori* and intestinal parasitic infections was assessed for the first time among Lebanese hospitalized children and adolescents. A moderate prevalence of *H. pylori* and intestinal parasite co-infection was observed and associated with the low educational level of the participants’ mothers. In regards to the clinical manifestations reported in this study, gastric reflux was significantly associated with *H. pylori* infection and severe diarrhea showed a significant relationship with intestinal parasitic infection. This study could provide valuable insights into the complexity of the interconnected relationship between enteric pathogens and how they can promote each other’s persistence in the gut, and thus affect child health and development.

### Ethics and consent

This cross-sectional hospital-based study was conducted in two hospitals located in Northern Lebanon from October 2023 to December 2023. The institutional review board (IRB) committee at Beirut Arab University approved this study on October 2023 (2023-H-0154-HS-R-0548), in accordance with the Declaration of Helsinki. Written informed consent was obtainedfrom parents or legal guardians before participation in the study.

## Data Availability

Figshare: Raw data collected from patients enrolled in this study: Mina, Sara (2024). RAW Data_Children_HP and Parasites.sav. figshare. Dataset. https://doi.org/10.6084/m9.figshare.25292653.v1. Data are available under the terms of the Creative Commons Attribution 4.0 International license (CC-BY 4.0). Figshare: Questionnaire Concomitant infection of Helicobacter pylori and intestinal parasites, https://doi.org/10.6084/m9.figshare.25690047.v1 Data are available under the terms of the Creative Commons Attribution 4.0 International license (CC-BY 4.0).
