# Supplementary material for: Concomitant Infection of Helicobacter pylori and Intestinal Parasites: Burden, Sociodemographic and Clinical Characteristics in Hospitalized Children and Adolescents in Northern Lebanon
Source: F1000Res. 2024 Oct 15;13:500. Originally published 2024 May 17. [Version 2] doi: 10.12688/f1000research.148550.2 (PMC11809640; doi:10.12688/f1000research.148550.2)
Supplement: Peer review history

# Reviewer report: Reviewer response for version 2

**DOI:** 10.5256/f1000research.172946.r345967

**Published:** 2024-12-26

**Authors:** Muinah Fowora

**Affiliations:** 1 Nigerian Institute of Medical Research, Lagos, Nigeria

Thank you for this interesting article. I understand the amount of work you have put into this article.

 The manuscript "Concomitant Infection of *Helicobacter pylori *and Intestinal Parasites: Burden, Sociodemographic and Clinical Characteristics in Hospitalized Children and Adolescents in Northern Lebanon" describes the prevalence of *H. pylori* and intestinal parasite co-infection in children 1 to 15 years old in Lebanon and also identifies the associated risk factors of these infections.

 The author clearly explained the study's objective and provided adequate explanations of the methodology. However, I find an issue with the statistical analysis employed in the study.

 Considering that the authors collected information on multiple variables, a bivariate analysis like Chi-square is not adequate. The authors should carry out multinomial logistic regression analysis for this study. This will identify significant risk factors while accounting for confounding variables.

 For example, the concluding statement in the results section states, “Children and adolescents of mothers with primary education or lower exhibited a significantly elevated likelihood of HP-IP compared to those born to mothers with secondary education or higher ( *p* = 0.025). Furthermore, patients from households that reported never washing vegetables before eating were significantly more likely to be infected with *H. pylori* only ( *p* = 0.010). Surprisingly, children and adolescents from households that reported washing hands correctly after defecating were significantly more likely infected with intestinal parasites only ( *p* = 0.046)." The authors need to check if these variables are still significant after accounting for other variables.

Is the work clearly and accurately presented and does it cite the current literature?

Yes

If applicable, is the statistical analysis and its interpretation appropriate?

Partly

Are all the source data underlying the results available to ensure full reproducibility?

Yes

Is the study design appropriate and is the work technically sound?

Yes

Are the conclusions drawn adequately supported by the results?

Partly

Are sufficient details of methods and analysis provided to allow replication by others?

Yes

Reviewer Expertise:

Helicobacter pylori infection, bacterial pathogenesis, antimicrobial resistance.

I confirm that I have read this submission and believe that I have an appropriate level of expertise to confirm that it is of an acceptable scientific standard, however I have significant reservations, as outlined above.

# Reviewer report: Reviewer response for version 2

**DOI:** 10.5256/f1000research.172946.r331970

**Published:** 2024-10-24

**Authors:** Steven F Moss, Suvithan Rajadurai

**Affiliations:** 1 Brown University, Rhode Island, USA; 2 Brown University, Providence, Rhode Island, USA

The authors have satisfactorily replied to points 1,5, and 6 but the methodological details of points 2,3, and 4 have not been directly placed into the text of the manuscript. We would suggest adding the following comments verbatim into the manuscript:

 1. "To assess the level of handwashing practices, the participants or their parents were asked about using water only or water and soap, the duration of handwashing, and how often they wash their hands after toilet. In addition, they were asked about washing vegetables under running water and rubbing them with their hands to remove surface microorganisms."

 2. "According to the manufacturer's guidelines for the H. PYLORI QUICK CHEK™ kit (TECHLAB, USA), watery stools were not collected for the stool antigen test (SAT). To address this, patients with severe diarrhea were asked to provide stool samples immediately after the episode had subsided." Additionally, would suggest commenting on the limitation this poses to the study in the discussion section

 3. "Patients with culture-confirmed bacterial gastroenteritis or diagnosed viral infections, such as rotavirus or adenovirus, identified through routine hospital investigations, were excluded from the study."

Is the work clearly and accurately presented and does it cite the current literature?

Partly

If applicable, is the statistical analysis and its interpretation appropriate?

Partly

Are all the source data underlying the results available to ensure full reproducibility?

Yes

Is the study design appropriate and is the work technically sound?

Partly

Are the conclusions drawn adequately supported by the results?

Partly

Are sufficient details of methods and analysis provided to allow replication by others?

Partly

Reviewer Expertise:

Helicobacter pylori, susceptibility testing, NGS

We confirm that we have read this submission and believe that we have an appropriate level of expertise to confirm that it is of an acceptable scientific standard.

# Reviewer report: Reviewer response for version 1

**DOI:** 10.5256/f1000research.162876.r318918

**Published:** 2024-09-19

**Authors:** Steven F Moss, Suvithan Rajadurai

**Affiliations:** 1 Brown University, Rhode Island, USA; 2 Brown University, Providence, Rhode Island, USA

In this study by Mina et. al the prevalence and associations between intestinal parasitic infection and H. pylori infection is examined among a cohort of children aged 1-15 years hospitalized with gastrointestinal symptoms in Northern Lebanon.  A modest association between H. pylori infection and intestinal parasites is demonstrated, suggesting similar modes of transmission. Some other positive clinical correlations are reported, suggesting etiological factors. I have the following concerns: 

1. The study involved parasite testing, interviews with patients/and or their family members etc beyond that necessary for routine investigation of the mainly upper and non-specific GI symptoms. Was IRB approval obtained for these extra elements? How were the extra tests and interviews funded?

1. Details regarding how the histories of vegetable washing, handwashing practices etc are obtained is lacking. Were the patients quizzed, or were there relatives? What questions were asked specifically? 

1. Within the methods section the authors describe that they collected fresh stool samples for H. pylori and parasite evaluation. However, some of the patients had diarrhea which could dilute the samples, leading to false negative results. This may explain why the H. pylori prevalence in this cohort is lower than found in many other studies from the middle East. There are no controls, e.g patients without gastrointestinal symptoms, that could shed light upon this issue.

1. The protocol for pathogen testing is not well-defined. Which bacterial and protozoal species were evaluated specifically. Were stools cultured for salmonella, shigella, etc? How were viral infections excluded?

1. The authors should discuss the clinical implications of their findings 

1. As a cross sectional study there are intrinsic limitations including lack of ability to determine causality that should be outlined within the manuscript. Generalizability is also a concerns, as well as the methodological issues discussed above. I suggest the authors include the strengths and limitations of their study design within the discussion or conclusion section 

Overall, I would recommend against acceptance until the above recommendations and comments are addressed.

Is the work clearly and accurately presented and does it cite the current literature?

Partly

If applicable, is the statistical analysis and its interpretation appropriate?

Partly

Are all the source data underlying the results available to ensure full reproducibility?

Yes

Is the study design appropriate and is the work technically sound?

Partly

Are the conclusions drawn adequately supported by the results?

Partly

Are sufficient details of methods and analysis provided to allow replication by others?

Partly

Reviewer Expertise:

Helicobacter pylori, susceptibility testing, NGS

We confirm that we have read this submission and believe that we have an appropriate level of expertise to state that we do not consider it to be of an acceptable scientific standard, for reasons outlined above.

# Response

**Published:** 2024-10-10

**Authors:** Sara MINA

Dear Dr. Moss,

 We sincerely appreciate your efforts in refining our manuscript. Your insightful comments were highly valuable and helped us significantly improve the quality of our work. Every comment and concern raised has been meticulously addressed, and we have made all the necessary modifications. These changes have been comprehensively tracked in review mode for your reference. We are hopeful that with these revisions, the manuscript now fulfills the requirements for publication in the F1000Research journal.

**Comment #1:**

 The study involved parasite testing, interviews with patients/and or their family members etc beyond that necessary for routine investigation of the mainly upper and non-specific GI symptoms. Was IRB approval obtained for these extra elements? How were the extra tests and interviews funded?

**Response:** IRB approval for the entire study, including additional tests and interviews, was obtained from the Institutional Review Board (IRB) at Beirut Arab University (Approval No. 2023-H-0154-HS-R-0548). Furthermore, parental consent and child assent were secured before participation. All participants provided informed consent, confirming their voluntary involvement in the study. There was no incentive for the interviews, and the extra tests were performed as a part of the routine hospital investigations.

**Comment #2:**

 Details regarding how the histories of vegetable washing, handwashing practices etc are obtained is lacking. Were the patients quizzed, or were there relatives? What questions were asked specifically?

**Response:** Thank you for these critical questions. The patients were interviewed in the presence of their parents. Adolescents responded by themselves, and the parents responded on behalf of younger participants. To assess the level of handwashing practices, the participants or their parents were asked about using water only or water and soap, the duration of handwashing, and how often they wash their hands after toilet. In addition, they were asked about washing vegetables under running water and rubbing them with their hands to remove surface microorganisms.

**Comment #3:**

 Within the methods section the authors describe that they collected fresh stool samples for H. pylori and parasite evaluation. However, some of the patients had diarrhea which could dilute the samples, leading to false negative results. This may explain why the *H. pylori* prevalence in this cohort is lower than found in many other studies from the middle East. There are no controls, e.g patients without gastrointestinal symptoms, that could shed light upon this issue.

**Response:** Thank you for this valuable and insightful comment. The authors fully agree that watery stool samples could lead to a higher incidence of false negatives. According to the manufacturer's guidelines for the H. PYLORI QUICK CHEK™ kit (TECHLAB, USA), watery stools were not collected for the stool antigen test (SAT). To address this, patients with severe diarrhea were asked to provide stool samples immediately after the episode had subsided. The authors acknowledge that this may present a limitation in the study. We concur that future research should focus on asymptomatic subjects to explore similar correlations between *H. pylori* and intestinal parasites.

**Comment #4:**

 The protocol for pathogen testing is not well-defined. Which bacterial and protozoal species were evaluated specifically. Were stools cultured for salmonella, shigella, etc? How were viral infections excluded?

**Response:** Thank you for this important technical question. In this study, stool samples were not cultured for the isolation of *Salmonella* or *Shigella*. The primary focus of the study was on detecting *H. pylori* using the H. PYLORI QUICK CHEK™ kit, as well as identifying intestinal parasites through direct-light microscopy (DLM). Additionally, a modified Ziehl-Neelsen (MZN) staining technique at 1,000× magnification was used to detect coccidian parasites. Patients with culture-confirmed bacterial gastroenteritis or diagnosed viral infections, such as rotavirus or adenovirus, identified through routine hospital investigations, were excluded from the study.

**Comment #5:**

 The authors should discuss the clinical implications of their findings

**Response:** Thank you for this valuable comment. *H. pylori* is a known contributor to gastric ulcers, chronic gastritis, and potentially gastric cancer. However, when parasitic infections occur simultaneously, they can intensify or obscure these symptoms, making diagnosis more challenging. Clinicians may need to adopt a broader differential diagnosis when evaluating gastrointestinal issues, ensuring both bacterial and parasitic infections are considered. Moreover, the interaction between *H. pylori* and parasitic infections may impact treatment options. For instance, standard *H. pylori* therapies like antibiotics might be less effective if parasitic infections are not treated concurrently. Administering antiparasitic therapies alongside *H. pylori* eradication could improve outcomes but may also introduce concerns about drug interactions or resistance. These findings may shape screening protocols, emphasize the importance of integrated treatment strategies, and prompt the development of guidelines for managing co-infections in clinical practice.

**Comment #6:**

 As a cross sectional study there are intrinsic limitations including lack of ability to determine causality that should be outlined within the manuscript. Generalizability is also a concerns, as well as the methodological issues discussed above. I suggest the authors include the strengths and limitations of their study design within the discussion or conclusion section

**Response:** Thank you for this valuable feedback. To the best of our knowledge, this study is the first to provide novel data on the association between *H. pylori* and intestinal parasites in a Lebanese pediatric population attending tertiary care hospitals. This represents a key strength of the study, especially given the limited epidemiological research on *H. pylori* in Lebanon. However, we acknowledge that the findings may not be fully generalizable to the broader Lebanese population. The cross-sectional design of the study limits the ability to establish strong causal relationships, and the recruitment of hospitalized patients with gastrointestinal symptoms may have affected internal validity. These limitations have been discussed in the discussion section, as suggested, and are reflected in the track changes.

# Reviewer report: Reviewer response for version 1

**DOI:** 10.5256/f1000research.162876.r284870

**Published:** 2024-06-13

**Authors:** Marwa Gouda

**Affiliations:** 1 National Liver Institute, , Menoufia University, Menoufia, Egypt

Thank you for the opportunity to review the manuscript titled “Concomitant Infection of Helicobacter pylori and Intestinal Parasites: Burden, Sociodemographic and Clinical Characteristics in Hospitalized Children and Adolescents in Northern Lebanon”. I would like to appreciate the authors' effort to undertake this valuable task. I have some concerns that may help to improve the manuscript.

- The authors reported limited research in their subject matter, however by searching some authors previously documented this subject with the same details. It would be better to concise limited research to the locality of research and add data concerning the prevalence of the examined pathogen in the research locality.
- It would be beneficial to have a more detailed background on why the concomitant infection of H. pylori and intestinal parasites specifically is of interest.
- The procedures used in stool examinations are limited and inadequate to diagnose all intestinal parasites. Have the authors performed any other techniques or special stains in diagnosis??
- Consider whether there should be more detail on the specific laboratory techniques used for detecting H. pylori and intestinal parasites.
- It might be useful for the authors to hypothesize why certain sociodemographic factors (e.g., mother's education level) are associated with higher rates of co-infection.
- The study's limitations are not explicitly mentioned in the provided snippet. Please, discuss potential limitations and their impact on the findings.
- The authors should provide suggestions for future research based on their findings.

Is the work clearly and accurately presented and does it cite the current literature?

Yes

If applicable, is the statistical analysis and its interpretation appropriate?

Yes

Are all the source data underlying the results available to ensure full reproducibility?

Yes

Is the study design appropriate and is the work technically sound?

Partly

Are the conclusions drawn adequately supported by the results?

Partly

Are sufficient details of methods and analysis provided to allow replication by others?

Partly

Reviewer Expertise:

parasitology, infectious diseases

I confirm that I have read this submission and believe that I have an appropriate level of expertise to confirm that it is of an acceptable scientific standard, however I have significant reservations, as outlined above.

# Response

**Published:** 2024-10-10

**Authors:** Sara MINA

Dear Dr. Gouda,

 We sincerely appreciate your efforts in refining our manuscript. Your insightful comments were highly valuable and helped us to significantly improve the quality of our work. Every comment and concern raised has been meticulously addressed, and we have made all the necessary modifications. These changes have been comprehensively tracked in review mode for your reference. We are hopeful that with these revisions, the manuscript now fulfills the requirements for publication in the F1000Research journal.

**Comment #1:**

 The authors reported limited research in their subject matter, however by searching some authors previously documented this subject with the same details. It would be better to concise limited research to the locality of research and add data concerning the prevalence of the examined pathogen in the research locality.

**Response:** Thank you for your valuable comments and suggestions. We have now included additional references related to the prevalence of the pathogen in the research locality, as recommended (Naous et al., 2007; Al Kirdy et al., 2020). You may view the revisions made in the track changes mode. The Naous et al. (2007) study was initially omitted from the manuscript because it is an older fecoprevalence study that focused solely on the prevalence of H. pylori in children, without addressing any concurrent intestinal parasites. However, we have reconsidered its relevance and included it as per your suggestion.

**Comment #2:**

 It would be beneficial to have a more detailed background on why the concomitant infection of *H. pylori* and intestinal parasites specifically is of interest.

**Response: **We appreciate the valuable feedback provided by the reviewer. Indeed, the authors have not highlighted enough the interest behind the concomitant infection of *H. pylori* and intestinal parasites. Co-infections can modulate disease dynamics through synergistic or antagonistic interactions. Interestingly, the co-existence of *H. pylori *and intestinal parasites may alter the disease severity and skew host immune responses (Jaka and Smith, 2024; Krzyżek and Gościniak, 2017). Changes have been implemented and tracked under review mode.

**Comment #3:**

 The procedures used in stool examinations are limited and inadequate to diagnose all intestinal parasites. Have the authors performed any other techniques or special stains in diagnosis??

**Response: **Thank you for your valuable comments. The authors acknowledge the importance of utilizing the most appropriate techniques for diagnosing intestinal parasites. However, given the available resources in our research laboratory and the project’s budget constraints, we primarily relied on wet mount preparations in normal saline and direct-light microscopy (DLM) for detecting intestinal parasites, observing either partial or whole parasite structures. To ensure accuracy, all examinations were conducted in duplicate by two experienced microbiologists. Additionally, a modified Ziehl-Neelsen (MZN) staining technique at 1,000× magnification was employed to detect coccidian parasites.

 For the detection of *H. pylori* in stool samples, we utilized the H. PYLORI QUICK CHEK™ kit (TECHLAB, USA), following established methodologies (Halland et al., 2021).

 Halland M, Haque R, Langhorst J, Boone JH, Petri WA. Clinical performance of the H. PYLORI QUIK CHEK™ and H. PYLORI CHEK™ assays, novel stool antigen tests for diagnosis of Helicobacter pylori. Eur J Clin Microbiol Infect Dis. 2021 May;40(5):1023-1028. doi: 10.1007/s10096-020-04137-7. Epub 2021 Jan 3. PMID: 33389260; PMCID: PMC7778841.

**Comment #4:**

 Consider whether there should be more detail on the specific laboratory techniques used for detecting *H. pylori *and intestinal parasites.

**Response: **Thank you for your suggestion. It was addressed in the previous response to comment #3.

**Comment #5:**

 It might be useful for the authors to hypothesize why certain sociodemographic factors (e.g., mother's education level) are associated with higher rates of co-infection.

**Response: **Thank you for your insightful comment. We agree that the relationship between a mother’s education level and higher rates of co-infection was not sufficiently explored in our initial discussion. We hypothesize that a mother’s education level can significantly influence hygiene practices within the household, which may impact the incidence of microbial infections in children. This hypothesis is supported by findings from Wangda et al. (2017), which demonstrated that a mother’s educational background is closely linked to her knowledge and traditional practices regarding the prevention and treatment of diseases in children (Wangda et al., 2017).

**Comment #6:**

 The study's limitations are not explicitly mentioned in the provided snippet. Please, discuss potential limitations and their impact on the findings.

**Response: **Thank you for highlighting this point. To the best of our knowledge, the present study is the first to report novel assessment and risk data on the association between *H. pylori* and intestinal parasites in a Lebanese pediatric population attending tertiary care hospitals. However, the findings may not be generalizable to the Lebanese population. The cross-sectional design did not allow for drawing causal conclusions. In addition, the recruitment of hospitalized patients with gastrointestinal symptoms may affect internal validity. Adding to this, the authors agree that using more appropriate techniques to identify intestinal parasites with more precision could be one of the limitations to be considered in future studies.

**Comment #7:**

 The authors should provide suggestions for future research based on their findings.

**Response: **Thank you for your suggestion. In this study, we focused on hospitalized children and adolescents in Northern Lebanon. Moving forward, we plan to expand this research to include asymptomatic children and adults, given that *H. pylori *infection is often asymptomatic and widespread. To build on our findings, future large-scale, multicenter epidemiological studies with appropriate control groups are needed. These studies should employ a variety of diagnostic methods to further validate our results and investigate the mechanistic interactions between *H. pylori *and specific parasitic infections. Additionally, metagenomic analyses of stool samples could help identify distinct pathogenic profiles (including parasites, viruses, and fungi) and their associations with *H. pylori* virulence factors, such as CagA, VacA, babA, and others.
